# IgE Aggravates the Senescence of Smooth Muscle Cells in Abdominal Aortic Aneurysm by Upregulating LincRNA-p21

**DOI:** 10.14336/AD.2018.1128

**Published:** 2019-08-01

**Authors:** Wenjun Guo, Ran Gao, Wei Zhang, Weipeng Ge, Meng Ren, Bolun Li, Hongmei Zhao, Jing Wang

**Affiliations:** State Key Laboratory of Medical Molecular Biology, Institute of Basic Medical Sciences, Chinese Academy of Medical Sciences, Department of Pathophysiology, Peking Union Medical College, Beijing, China

**Keywords:** Asthma, IgE, Abdominal aortic aneurysms, Senescence, lincRNA-p21, p21

## Abstract

Immunoglobulin E (lgE) activates immunity by binding to mast cells and basophils. It is well-known that IgE and its receptor, FcɛR1, play a key role in the development of airway inflammation and remodeling in allergic asthma. Recent studies show that IgE also plays an important role in abdominal aortic aneurysm (AAA) pathogenesis. However, the mechanism by which IgE promotes AAA remains unclear. Here we report that in our mouse model, asthma-induced high level of IgE aggravated AAA, but IgE lost this effect on AAA in FcɛR1^-/-^ mice. Our in vitro study revealed that IgE induced smooth muscle cell senescence via upregulating lincRNA-p21 against p21 without altering expression of p53. By this mechanism, IgE accelerated AAA in ApoE^-/-^ mice, which was blocked by knockdown of lincRNA-p21 in both vitro and vivo. This study suggests that IgE actives the lincRNAp21-p21 pathway to induce SMC senescence, which contributes to the formation of AAA, and lincRNA-p21 is a potential therapeutic target for AAA aggravated by asthma.

Abdominal aortic aneurysm (AAA) in which the abdominal aortic wall degenerates and expands more than 50% of the normal vascular diameter, is a pathological state influenced by multiple factors. The end point of AAA is rupture and bleeding, leading to the sudden death of patients. Its mortality rate is as high as 85% with poor prognosis if it ruptures [1]. Current studies have shown that AAA is an inflammatory disease, such as IL-6, which is one of the most abundantly expressed cytokines in AAA tissue [2]. The aging of smooth muscle cells (SMCs) would lead to changes in the function of the vessel wall and the formation of AAA, besides, Dr. Chen demonstrated that SIRT1 acted as a novel molecular link that retards vascular senescence and inflammation in SMCs [3]. SMC, as the principal resident cells of aortic wall, maintain essentially vessel structure by secretion of extracellular matrix. In addition, upregulated matrix metalloproteinases (MMPs) could effectively degrade the extracellular matrix and damage the vascular wall structure, causing AAA [4]. SMCs are unique to induce repair in the damaged vessel and this makes them a potential target for further study.

Immunoglobulin E (lgE) is the least abundant immunoglobulin in normal human serum, which causes type I hypersensitivity when it is upregulated [5]. IgE can bind to mast cells and basophils to activate immune functions, causing the body to enter the allergen specific sensitization state. As the main cause of the allergic inflammatory reaction, IgE participates in bronchial obstruction, and has a role in allergic asthma exacerbations [6]. Allergic asthma is the most common asthma phenotype, which is a chronic airway inflammation with a variety of cells involved in, especially mast cells, eosinophils, and T lymphocytes, of which the pathogenesis is elevated levels of specific IgE [7]. Mast cells are activated by cross-linking between Antigen (Ag)-specific IgE and FceRI on their membrane, secreting a variety of mediators, which contribute to airway inflammation and remodeling in allergic asthma [8].

Long chain non-coding RNA (lincRNA) is one type of RNA molecules between 200~100000 nucleotides without protein coding function. LincRNAs can be situated in the nucleus or cytoplasm [9], and are involved in a wide range of biological processes [10]. Moreover, lncRNAs have been reported as important players in the initiation and development of diseases, particularly cardiovascular diseases, including myocardial infarction, cardiomyopathy, heart failure, and atherosclerosis [11]. For instance, during cardiac development, Braveheart (Bvht), as a lincRNA, regulates gene activation or suppression to activate stem cell differentiation [12]. Another lincRNA, ANRIL, locating in 9p21 has been proven to lead to the inhibition of vascular smooth muscle cell growth, and in turn increase the risk of atherosclerotic vascular diseases [13]. Besides, lincRNAs regulate gene transcription and the fate of post-transcribed mRNA, which affects a broad range of age-associated physiological and pathological conditions, including cardiovascular diseases and cellular senescence [14]. Moreover, during atherosclerosis, lincRNA-p21 represses cell proliferation, neointima formation and induced apoptosis by directly binding to mouse double minute 2 (Mdm2), which leads to P53 release from Mdm2 to enhance P53 activity [15]. However, whether and how lncRNA affects AAA remains unclear.

We previously found that asthma upregulated IgE, aggravated the formation of AAA. In this this study, we investigated the effect of IgE on the senescence of smooth muscle cell (SMC) contributing to AAA, and the underlying mechanism in vivo and in vitro. Our study suggests that IgE promotes the development of AAA in a large part by inducing SMC senescence via a pathway composed of lincRNAp21-p21.

## MATERIALS AND METHODS

### Animal model

All animal experiments were performed with the approval of the Research Ethics Committee of Peking Union Medicine College. All mice were 8-week-old male mice. Mice with weight of 20-22g were maintained in a temperature and humidity-controlled room with a 12 h light-dark cycle. Mice had free access to tap water and standard mouse chow ad libitum. We crossbred FcɛR1α^-/-^mice (C57BL/6, N9, provided by Marie-Helene Jouvin and Jean-Pierre Kinet, Harvard Medical School) with ApoE^-/-^mice (C57BL/6, N11, The Jackson Laboratory, Bar Harbor, ME) to generate ApoE^-/-^FcɛR1α^-/-^mice. All mice used in this study were littermates in a syngeneic C57BL/6 background.

ApoE^-/-^ littermates were randomly assigned to the asthma group (n=20) or control group (n=20). Briefly, the experimental mice were sensitized by intraperitoneal injection (i.p.) of 50 μg ovalbumin with alum adjuvant[16] on day 0, 7 and 14, while the control mice received i.p. saline with alum. From day 21 onward, the mice were exposed to aerosolized 3.75% OVA (allergen exposure) or PBS (sham exposure) 30 min/day for 3 successive days.

To induce AAA in ApoE^-/-^ and ApoE^-/-^FcɛR1α^-/-^mice, on day 24, anesthetized (200 mg/kg ketamine, 10 mg/kg xylazine, intraperitoneal injection) mice were infused with 1000 ng/kg/min Ang-II (A9525-50MG Sigma-Aldrich, St. Louis, MO) subcutaneously delivered by Alzet model 2004 osmotic minipumps (DURECT Corp., Cupertino, CA) for 28 days while mice consumed a high-fat diet (C12108; Research Diets, Inc., New Brunswick, NJ) and continue to exposed to aerosolized 3.75% OVA (allergen exposure) or PBS (sham exposure) 30 min/day, once every other day for 28 days.

For lentivirus treated mice, the asthma and AAA induced ApoE^-/-^ littermates were randomly assigned to the si-mlincRNA-p21 group or control group. The experimental mice were injected with concentrated viral particles in the lateral tail veins on day 20, 27, 34, 41 and 48.

### Cytokine and lipoids in serum

Mice were sacrificed with anesthetized narcosis, followed by blood collection via cardiac puncture. Plasma IL-6 (Cat# 85-88-7064-77), TNF-α (Cat# 85-88-7324-22), IFN-γ (Cat# 85-12-7311-82), MCP-1 (Cat# 85-88-7391-77) and IgE (Cat# 500840) levels were determined by ELISA according to the manufacturer’s protocol (eBiosciences, San Jose, CA). After exsanguinations via cardiac puncture, bronchoalveolar lavage fluid (BALF) was collected (0.8 ml saline, two times) by tracheal intubation.

### Mouse lesion characterization

The death was confirmed by cervical dislocation. Allergic inflammatory responses were assessed in situ by lung histopathology and ex vivo by eosinophils counts of BALF. The suprarenal maximal aortic diameter of each aneurysm was measured after the peri-adventitial tissue was carefully dissected from the aortic wall. AAA incidence was defined by increase of suprarenal maximal aortic diameter greater than 50% of the mean value from same-age mice that received saline alone, according to previously reported methods. The percentage of AAA incidence and post-Ang-II infusion mortality rate per group were determined at the end of the experiment. All procedures were approved by the Animal Care and Use Committee of Peking Union Medical College.

### Mouse aortic tissue immunohistochemical analysis

Aorta segments for immunohistochemistry were cut at the maximal suprarenal outer aortic diameter and embedded vertically with optimal cutting temperature (OCT) compound, and at least 10-15 serial frozen sections covering the maximal dilated aorta were prepared for immunohistochemical analysis. For those with similarly enlarged AAA diameter throughout the thoracic-abdominal aortas, we selected the segment at approximately the same distance from the renal artery as those of others with maximal AAA expansions. In cases with AAA lesions at multiple locations, we selected the largest lesion as close as possible to the same distance from the renal artery as those of others with maximal AAA expansions. Slides of each sample from identical levels were used for staining with each antibody. Serial cryostat cross-sections (6 µm) were used for immunostaining for macrophages (Mac-3, 1:2,000, Cat# 553322, BD Biosciences), T cells (CD4, 1:90, Cat# 553043, BD Biosciences ), dendritic cells (MHC-II, 1:250, Cat# 556999, BD Biosciences), MCP-1 (1:200, ab25124, Abcam), TNF-α (1:200, ab6671, Abcam), IL-6 (1:200, ab191194, Abcam), elastin (Verhoeff-van Gieson, Sigma-Aldrich), collagen (solarbio), Ki67 (cell proliferation marker, 1:200, ab66155, Abcam) and CD31 (angiogenesis marker,1:1,500, Cat# 550274, BD Biosciences). Lesion apoptotic cells were determined with the *in situ* apoptosis detection kit according to the manufacturer’s instructions (Roche). Elastin degradation and media SMC accumulation were graded according to the grading keys described previously. CD4^+^T cells, macrophage, mast cells and apoptotic-positive cells were counted blindly. Unlike aortic cross sections from fixed tissues that allowed counting of most individual immunopositive signals, we prepared aortic cross sections from unfixed abdominal aortas optimized for immunohistochemical study, but not for reliable enumeration of all individual immuno-positive cells. We therefore measured macrophage-positive, MHC-II-positive, MCP-1-positive and collagen-positive areas using computer-assisted image analysis software (Image-Pro Plus; Media Cybernetics, Bethesda, MD). Ang-II-induced AAAs often shown regions of disrupted media, we calculated AAA lesion areas from regions with both intact and fragmented media.

**Table 1:** Primer Sequences.

Gene Name	Primer Sequence (5’-3’)	GenBank code
**hlincRNA-p21 forward**	GCTGCTGAAGTAGGAGCTTT	KU881768.1
**hlincRNA-p21 reverse**	GGATTCTGCTGATTCCAGTG	
**Human p21 forward**	CATGTGGACCTGTCACTGTCTT	NM_001291549
**Human p21 reverse**	GCTTCCTCTTGGAGAAGATCAGC	
**Human p53 forward**	TCATCACACTGGAAGACTCCAG	NM_001126118
**Human p53 reverse**	GCTGGTGTTGTTGGGCAGT	
**Human GAPDH forward**	TCAACGACCACTTTGTCAAGCTCA	NM_001289745
**Human GAPDH reverse**	GCTGGTGGTCCAGGGGTCTTACT	
**mlincRNA-p21 forward**	CCACTCGCTTTCCATTTCCC	NR_036469
**mlincRNA-p21 reverse**	AACTGGAGACGGAATGTCTCAT	
**Mouse p21 forward**	TCGCTGTCTTGCACTCTGGTGT	NM_007669
**Mouse p21 reverse**	CCAATCTGCGCTTGGAGTGATAG	
**Mouse p53 forward**	GCGTAAACGCTTCGAGATGTT	NM_011640
**Mouse p53 reverse**	TTTTTATGGCGGGAAGTAGACTG	
**Mouse GAPDH forward**	AGGTCGGTGTGAACGGATTTG	NM_001289726
**Mouse GAPDH reverse**	TGTAGACCATGTAGTTGAGGTCA	

### RNA isolation and Real-Time Polymerase Chain Reaction

Total RNAs were extracted from cells and tissues using TRIzol reagent (Invitrogen, Carlsbad, CA). The RNA samples were then treated with a RNase-free DNase (Ambion, Carlsbad, Cal) to remove genomic DNA contaminants. Equal amounts of RNA were reverse transcribed, and quantitative PCR was performed in a single-color real-time polymerase chain reaction (RT-PCR) detection system (Bio-Rad, Hercules, CA, USA). The lincRNA-p21 level and mRNA levels of p21 and p53 were normalized to those of housekeeping gene-GAPDH.

### Western blotting analysis

Cells were collected by centrifugation (700 ×g for 10 min at 4°C) and lysed in RIPA. The protein concentration was determined using the Lowry method with bovine serum albumin (BSA) as a standard (Pierce, Holmdel, NJ, US). Equal amounts of protein were separated by sodium dodecyl sulfate-polyacrylamide gel electrophoresis (SDS-PAGE) using 12% polyacrylamide gels. The protein was subsequently transferred onto a polyvinylidene difluoride membrane by electroblotting for 2 h at 260 mA. Membranes were blocked for 1 h in TBST/5% low-fat milk powder and incubated with the primary antibody overnight in Tris buffered saline-Tween (TBST) 5% BSA or 5% low-fat milk. The secondary antibody, coupled with horseradish peroxidase (HRP), was applied for 1 h at room temperature. Chemiluminescence detection was done with HRP juice (PJK) and a CCD camera. Densitometric signals were quantified using Quantity One Bioanalysis software (Bio-Rad, Hercules, CA, USA). The protein level of signaling molecules was expressed as folds of control. Specific antibodies used were purchased from abcam (anti-p21, ab109520, 1:1000 dilution; anti-p53, ab1101, 1:1000 dilution), and proteintech (anti-GAPDH, 10494-1-AP, Proteintech, 1:5000 dilution). The intensities of the protein bands were analyzed by ImageJ software.

### Cell culture and small-interfering RNA (siRNA) assay

HSMC were purchased from ScienCell Research Laboratories, Inc (Cat# 6110, San Diego, CA, USA). The HSMC cells were cultured in SMCM medium (ScienCell) containing 5% fetal bovine serum, 100 U/mL of penicillin, and 100 g/mL of streptomycin, at 37°C in 5% CO2. For the ex vivo experiments, HSMC were cultured in 6-well plates (6×10^5^ cells/well; DingGuo Biotech, Chongqing, China) with IgE in 2mL of SMCM at 37°C in 5% CO2 for the time given. siRNA designed for Human lincRNA-p21 (Sense: gcaugauuguucuggauug; antisense: aaauccagaacaaucaugcaa) was purchased from RIBOBIO (Guangzhou, china). siRNA transfection was carried out according to the manufacturer’s protocol. HSMC cells were transferred onto 6-well plates and cultured in SMCM supplemented with 2% fetal bovine serum. After growth for 24 h, cells at 80% adherence were treated with 1 mL of Opti-MEM I (Invitrogen, Carlsbad, CA, USA), with each well containing 100 nmol/L siRNA duplexes and 8 μL of Oligofectamine. Cells were washed exhaustively and harvested. siRNA efficiency of RNA relative expression was determined by RT-PCR (Table 1).

### Senescence Cells Histochemical Staining

Senescence Cells Histochemical Staining Kit was purchased from sigma (cs0030). Cells were stained according to the manufacturer’s instructions.

### Lentivirus production

For mlincRNA-p21 knockdown, the double-stranded siRNA templates specific to mlincRNA-p21 and control templates were cloned into a pol Ⅲ promoter lentivector (pSIH-H1, SI501A-1, System Biosciences, CA, USA) to generate lenti-si-mlincRNA-p21 and lenti-ctrl. The matching lentivirus packaging kit was purchased from System Biosciences (SBI, CA, USA) and used according to the manufacturer’s instructions. The harvested viral particles were concentrated by ultracentrifuge and used in subsequent experiments.

### Statistical analysis

The progression of AAA was calculated by individual linear regression analysis of AAA diameter over observation time allowing all observations to be included. Mouse and cell-culture data were presented as means± SEM. Because of relatively small sample sizes and skewed data distribution, we selected the non-parametric Mann-Whitney U test for paired data sets, and one-way ANOVA with post-hoc Bonferroni test was used for comparison among three or more groups to examine statistical significance for all data from cultured cells and experimental AAA. Fisher’s exact test was used to compare the differences in AAA incidence and post-Ang-II mortality. P<0.05 was considered statistically significant.

## RESULTS

### Asthma induced high IgE accelerated AAA formation via IgE receptor, FcɛR1

**Figure 1.**
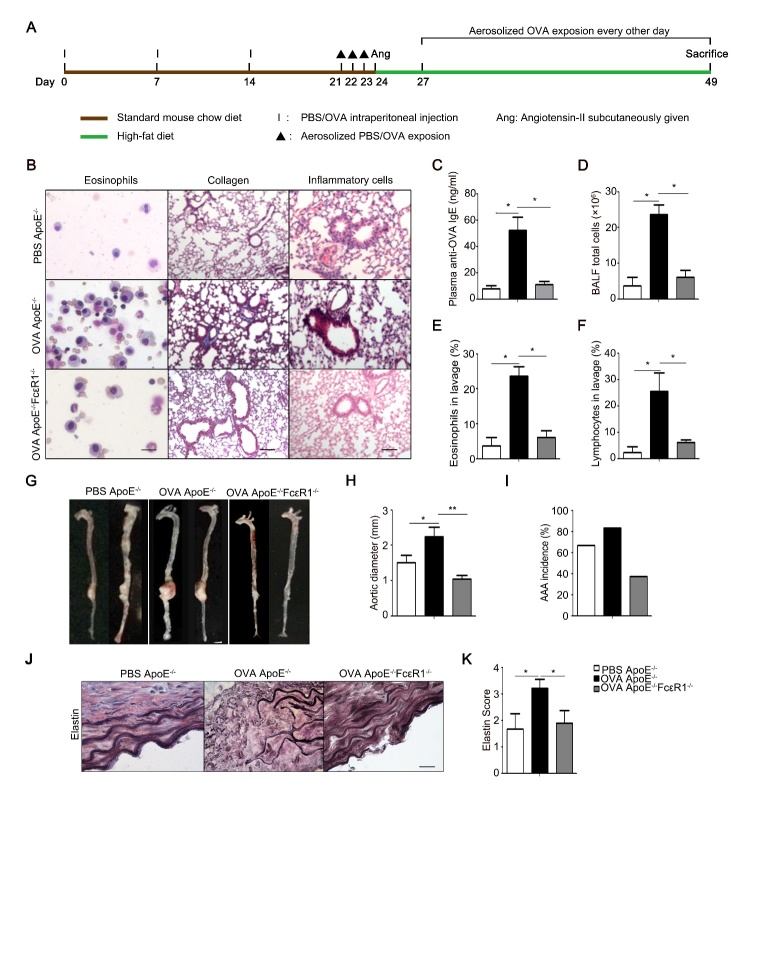
Effect of IgE receptor, FcɛR1, on asthma-accelerated AAA (**A**) Schematic diagram for the mouse model of asthma-accelerated AAA. (**B**) Representative pictures from Giemsa staining for bronchial-alveolar lavage fluid (BALF) cell, and Masson staining and H&E staining for lung tissue. Eosinophils Scale bars: 100μm. Collagen Scale bars: 50μm. Inflammation cells Scale bars: 50μm; (**C**) Anti-OVA IgE levels in serum; (**D**) Total number of cells in BALF; (**E**) Percentage of eosinophils in BALF; (**F**) Percentage of lymphocytes in BALF. (**G**) Images of mouse aorta of ApoE^-/-^mice treated with or without OVA, and ApoE^-/-^FcɛR1^-/-^ mice treated with OVA. (**H**) The average diameters of the AAA. (**I**) The incidence rate of AAA in ApoE^-/-^, OVA-treated ApoE^-/-,^ and OVA-treated ApoE^-/-^FcɛR1^-/-^ mice. (**J**) Representative staining for elastin at AAA lesions where the most severe elastin degradation occurred. Scale bars: 100μm. (**K**) Scores from the elastin degradation classification for AAA lesions of the mice with the indicated genotype. Data information: In (C-F, H, I, K), data are presented as mean ± SEM (n=15 per group). *P < 0.05; ^**^P<0.01

**Figure 2.**
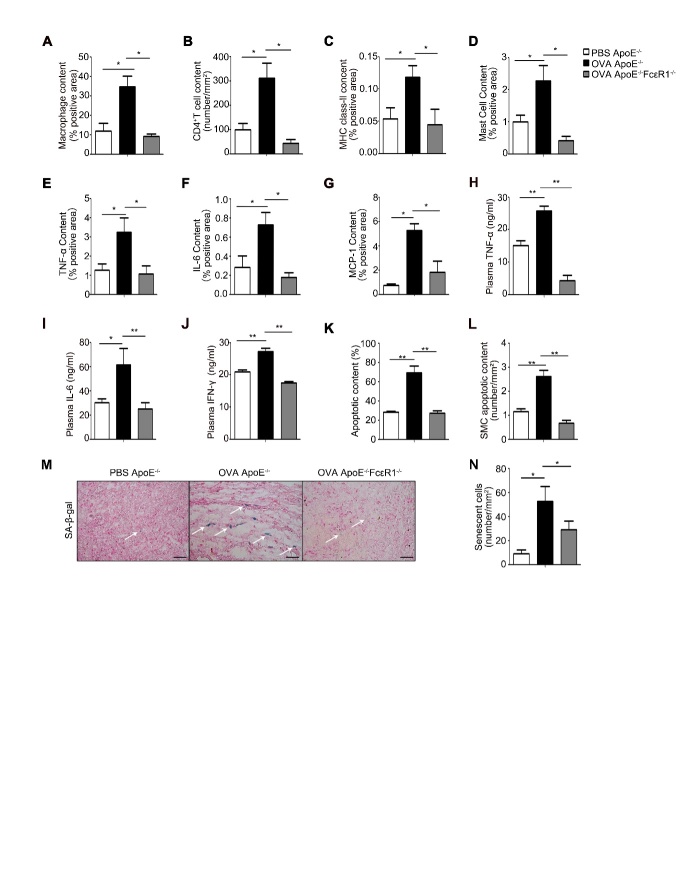
Inflammation and remodeling in AAA lesion (**A-G**) AAA lesion macrophage content (A); CD4^+^ T-cell content (B); major histocompatibility complex (MHC) class-II-positive area (C); mast cell content (D); and chemokine TNF-α-positive area (E); IL-6-positive area (F); and MCP-1-positive area (G) from ApoE^-/-^mice treated with or without OVA, and from OVA-treated ApoE^-/-^FcɛR1^-/-^ mice. (H-J) The expression of plasma inflammatory factors, TNF-α (H), IL-6 (I), and IFN-γ (**J**) in serum from PBS ApoE^-/-^, OVA ApoE^-/-^ and OVA ApoE^-/-^FcɛR1^-/-^ mice; (**K**) Statistic analysis of TUNEL staining for apoptotic cells in the AAA tissue from ApoE^-/-^mice treated with or without OVA, and from OVA-treated ApoE^-/-^FcɛR1^-/-^ mice. (**L**) Cell content of apoptotic vascular smooth muscle cell in AAA lesion from PBS ApoE^-/-^, OVA ApoE^-/-^ and OVA ApoE^-/-^FcɛR1^-/-^ mice. (**M**) Representative picture of β-galactosidase staining for senescent cells in AAA lesions. Scale bars: 25μm. (**N**) Counts for senescent cells/mm^2^ in the AAA lesions. Data information: Data are presented as mean ± SEM (n=15 per group). *P < 0.05; **P<0.01.

Allergic diseases, especially allergic asthma, are characterized by high level of IgE [17]. And, high serum IgE is associated with abdominal aortic aneurysm (AAA) patients and AngII-induced AAA mice [18]. To explore the relationship between asthma and AAA, ApoE^-/-^ mice were chosen to generate AngII-induced AAA mice for our animal models, and ovalbumin (OVA) was applied to induce asthma (Fig. 1A). As shown in figure 1B-F, OVA-treatment significantly increased the number of eosinophils in BLAF, the formation of collagen fibers, and the infiltration of inflammatory cells in pleural cavity in ApoE^-/-^, suggesting asthma was successfully induced with OVA. This OVA-induced asthma markedly aggravated AngII-induced AAA in FcɛR1^+/+^ mice but not in FcɛR1^-/-^ mice (Fig. 1G and H). Asthma also made FcɛR1^+/+^ mice more susceptible to AAA (Fig. 1I). As one of the most important signatures of human and animal AAA, elastin fragmentation in the aortic wall was examined in AAA mice with or without asthma. As shown in Figure 1J and K, asthma significantly enhanced elastin fragmentation in the aortic wall only in FcɛR1^+/+^ mice. These results indicate that asthma aggravates the occurrence and development of AAA, and this effect of asthma on AAA is dependent on IgE receptor, FcɛR1.

**Figure 3.**
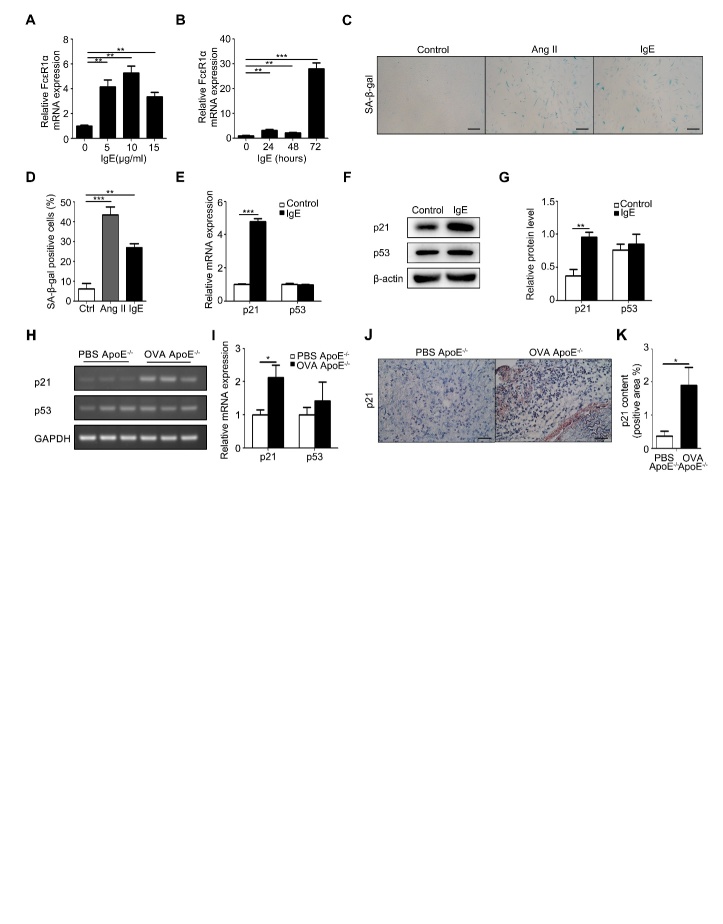
IgE effect on senescence of vascular SMCs in vitro and in vivo (**A**) RT-PCR detection of FceR1α expression at different doses of IgE stimulation. (**B**) RT-PCR detection of FceR1α expression at different times of IgE stimulation. (**C**) Representative images from β-galactosidase staining of human vascular smooth under the indicated conditions. Scale bars: 100 μm. (**D**) The statistics of the results from C. (**E**) RT-PCR detection of p21 and p53 mRNA expression in IgE-stimulated HSMCs. (**F**) Western blotting analysis of p21 and p53 expression in human vascular smooth muscle cells stimulated by IgE. (**G**) Statistical analysis on the relative intensities of p21 and p53 bands in the Western blotting of human vascular smooth muscle cells stimulated by IgE. (**H**) The expression of p21 and p53 mRNA in human vascular smooth muscle cells stimulated by IgE. (**I**) RT-PCR detection of p21 and p53 mRNA expression in human vascular smooth muscle cells stimulated by IgE (J) Representative pictures of p21 immunohistochemical staining in AAA lesions. Scale bars: 25μm. (**K**) The statistical analysis of p21 immunohistochemical staining in AAA lesions. Data information: Data are presented as mean ± SEM (animal tissues; n=15 per group). *P < 0.05; **P<0.01

### Deficiency of FcɛR1 prevented the pathological phenotypes of IgE-promoted AAA in vivo

To explore the role of FcɛR1 in the formation of IgE-promoted AAA, we examined the phenotype of AAA tissues in AAA mice with or without OVA-treatment, and OVA-treated AAA mice with or without FcɛR1. Since the formation of AAA is closely related to inflammation [19], we also investigated the effect of inflammation on the process of development of AAA. The analysis of immunohistochemical staining showed that the infiltration of macrophage (MAC-III), T-cell (CD4+), MHC class-II, and Mast cells in AAA tissues was significantly increased with OVA-treatment (Fig. 2A-D). And, the expression of major inflammatory factors (TNF-α, IL-6, and MCP-1) from the above cells were also significantly upregulated (Fig. 2E-G). In serum, TNF-α, IL-6 and IFN-γ were significantly richer in OVA-treated AAA mice than those in AAA mice without OVA-treatment (Fig. 2H-J). However, these aggravated inflammatory morphologies in AAA tissues did not appear in FcɛR1^-/-^ mice (Fig. 2A-J). These results suggest that asthma accelerates AAA pathology through IgE and its receptor.

Other than inflammation, aging and apoptosis of cells in the vascular wall also play a crucial role in the vascular remodeling. Therefore, we examined the apoptosis in the AAA tissues from the mice treated with or without OVA using TUNEL staining. As shown in Figure 2K, cell apoptosis in the AAA tissue was significantly enhanced by OVA treatment, which was especially pronouncing in smooth muscle cells (Fig. 2L). Compared with the group without asthma, the aortic senescence of asthmatic mice was more severe (Fig. 2M and N). However, OVA failed to enhance cell apoptosis and senescence in the AAA tissue from OVA-treated FcɛR1^-/-^ mice (Fig. 2K-N). These data indicate that IgE promotes the senescence and apoptosis of aortas to aggravate AAA pathology.

### IgE induced the senescence of HSMCs

To examine the role of IgE in AAA, human vascular smooth muscle cells (HSMC), a cell type widely used to study AAA *in vitro* [3], were treated with different concentrations of IgE (0, 5, 10, 15 μg/ml), and for different periods in the indicated time courses with 10μg/ml IgE which was the optimal concentration shown in the concentration curve (Fig. 3A). In response to this IgE treatment, expression of FcɛR1 mRNA peaked at 72h after the treatment (Fig. 3B). Under this condition, our SA-β-gal staining results showed that IgE was as potent as AngII (100nmol/ml), a known pro-senescence factor [20], in promoting the senescence of HSMC (Fig. 3C, D). RT-PCR for the classic senescence markers, p21 and its upstream factor, p53, showed that IgE significantly up-regulated p21, but not p53 (Fig. 3E-G). In AAA lesions in ApoE^-/-^ mice, OVA significantly enhanced expression of p21 without the change of p53 expression (Fig. 3H-K). These findings suggest that IgE induces HSMC senescence in vitro and vivo.

**Figure 4.**
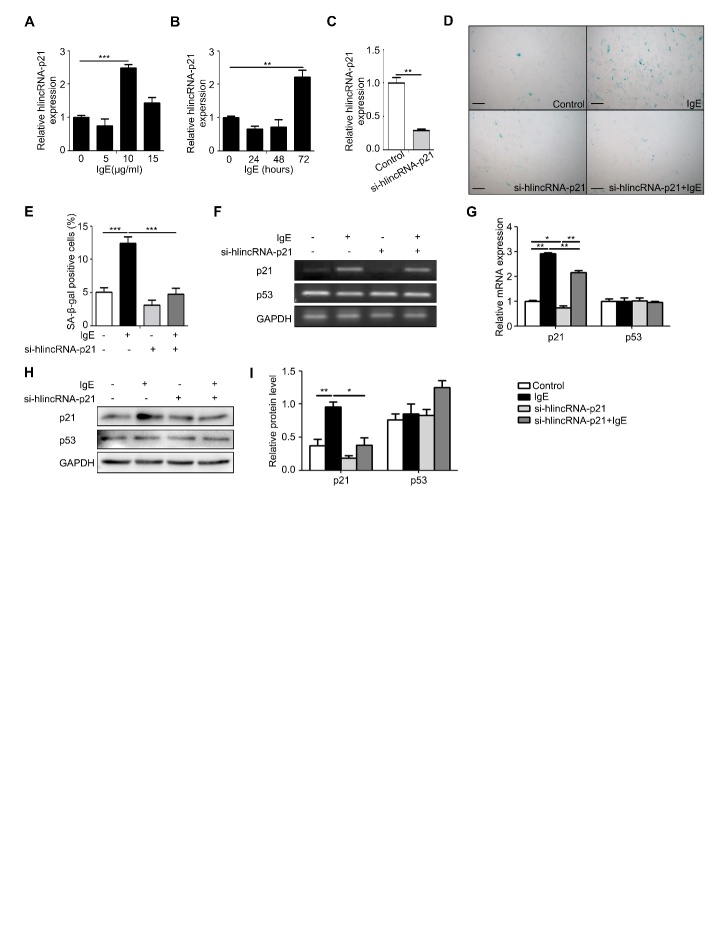
The effect of lincRNA-p21 on senescence of human vascular SMCs (**A**) The expression of lincRNA-p21 in human vascular smooth muscle cells stimulated by different doses of IgE. (**B**) The expression of lincRNA-p21 in human vascular smooth muscle cells stimulated by 10 μg/ml IgE at different time. (**C**) Detection of lincRNA-p21 expression in HSMC after transfectioned with lincRNA-p21 siRNA. (**D**) Representative images of HSMC β-galactosidase staining of control, IgE, si-lincRNAp21 and si-lincRNA-p21+IgE treatment groups. Scale bar: 100μm. (**E**) The senescent cell counts of HSMC after control, IgE, si-lincRNA-p21 or si-lincRNA-p21+IgE treatment. (**F**) PCR detection of p21 and p53 expression in HSMCs treated with IgE and si-lincRNA-p21. (**G**) Statistical analysis of p21 and p53 in HSMC treated with IgE and si-lincRNA-p21. (**H**) Western blot analysis of p21 and p53 in HSMC after IgE and si-lincRNA-p21 treatments. (**I**) Statistical analysis on the relative intensities of p21 and p53 protein expression in HSMC treated with IgE and si-lincRNA-p21. Data information: Data are presented as mean ± SEM. *P < 0.05; **P<0.01.

**Figure 5.**
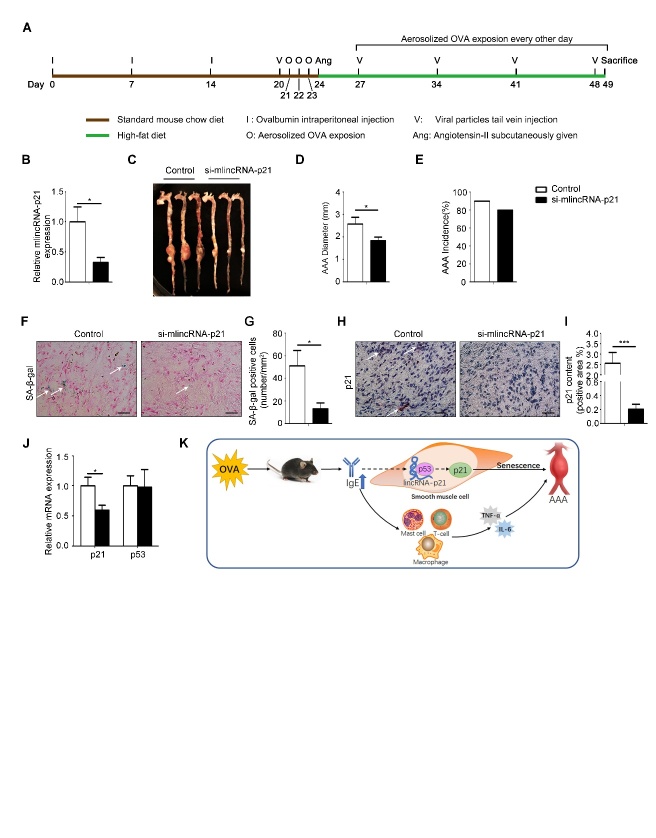
The effect of lincRNA-p21 on formation of AAA (**A**) Schematic diagram of animal model procedures. (**B**) Expression of lincRNA-p21 in AAA lesion of mice in control (n=8) and si-mlincRNA-p21 (n=5) groups. (**C**) Representative images of aortas from mice in control and si-mlincRNA-p21 groups of mice. (**D**) The average diameters of abdominal aortic aneurysm of two groups of mice. (**E**) The incidence rate of AAA in two groups of mice. (**F**) Representative pictures of β-galactosidase staining in AAA lesion. Scale bars: 25μm. (**G**) Senescent cells number per mm^2^ in AAA lesions. (**H**) Representative pictures of immunohistochemical staining for p21 in AAA lesions. Scale bars: 25μm. (**I**) The statistical analysis of p21 positive areas in immunohistochemical staining in AAA lesions. (**J**) RT-PCR detection of p21 and p53 mRNA expression in AAA lesions. (**K**) Schematic diagram of this article: IgE aggravates AAA mainly by upregulating lincRNA-p21 contributing to HSMC senescence. Data information: Data are presented as mean ± SEM. *P < 0.05; **P<0.01.

### IgE up-regulated the expression of lincRNA-p21 contributing to HSMC senescence

LincRNA-p21 is a long-chain non-coding RNA and is closely related to cardiovascular disease such as atherosclerosis [13]. As a direct transcription target for p53, and an integral part of the p53 pathway, it selectively down-regulates many p53 target genes by alternatively interacting with the p53 inhibitory complex [15]. Based on the above knowledge and our finding that IgE may promote p21 expression, we hypothesized that IgE might mediate lincRNA-p21 for regulating p21 to enhance SMCs senescence. To verify this hypothesis, we evaluated the effect of IgE on expression of lincRNA-p21 in HSMCs using different concentrations of IgE and for different periods in the indicated time course with 10 μg/ml IgE suggested by the concentration curve (Fig. 4A). The optimum stimulation for IgE to up-regulate lincRNA-p21 was 10 μg/ml IgE for 72h, which is the same as that for IgE to up-regulating FcɛR1 expression (Fig. 4A and B). To assess the role of lincRNA-p21 on HSMC senescence, we knocked down the expression of lincRNA-p21 using a small interfering RNA (si-hlincRNA-p21) in HSMC, which was validated by RT-PCR (Fig. 4C). This knockdown of lincRNA-p21 substantially prevented the IgE-induced senescence in HSMC examined by senescence-associated β-galactosidase staining (Fig. 4D and E). Meanwhile, both mRNA and protein of p21 were significantly down-regulated with the knockdown of lincRNA-p21 (Fig 4F-I). These data suggested that IgE induces senescence via up-regulating lincRNA-p21 in HSMCs.

### Inhibition of lincRNA-p21 rescued AAA in OVA-sensitized mice

To confirm the role of lincRNA-p21 in IgE-induced senescence, OVA-treated ApoE^-/-^ mice were infected with lentivirus-constructs (lentivirus containing control-siRNA or si-mlincRNA-p21) via tail vein injection (Fig. 5A). As shown in Figure 5B, the expression of lincRNA-p21 was depressed by the construct of si-mlincRNA-p21/lentivirus. Meanwhile the diameter of AAA was clearly reduced though there were no obvious difference in incidence (Fig. 5C-E). Consistent with the repression of lincRNA-p21 expression, significantly less p21 and SA-β-gal positive area was also presented in the stained AAA tissues (Fig. 5F-I). Concurrently, the mRNA level of p21 was also significantly lower than that in the mice infected with lentivirus containing the control siRNA. In either case, the level of p53 mRNA was not markedly altered (Fig. 5J). These data suggest that asthma-induced IgE promotes cell senescence in AAA via up-regulation of lincRNA-p21 and p21.

In conclusion, OVA induces asthma and high IgE in mice, which other than up-regulates inflammatory factors, also promotes the senescence of SMC by up-regulating the lincRNAp21-p21 pathway aggravating the development of AAA (Fig 5K).

## DISCUSSION

This study demonstrates that asthma aggravates AAA, and high-IgE promotes AAA pathology via FcɛR1. The injury of smooth muscle cell is one of the main causes of AAA. This report for the first time illustrates that high-IgE up-regulates LincRNA-p21 contributing to the senescence of SMC in AAA. In this event, IgE-FcɛR1 signaling up-regulates p21 expression in SMC through activation of LincRNA-p21. Knockdown of LincRNA-p21 in asthma mice markedly attenuates AAA. Importantly, *in vivo* expressed si-LincRNA-p21 efficiently represses the expression of senescence gene, p21, and attenuates the development of AAA in mice. Our findings support the role of IgE in regulation of AAA and indicate LincRNA-p21 as a potential therapeutic target for managing AAA.

As known, OVA can induce asthma with high IgE levels in vivo, which have been proved to aggravate AAA[18]. In 2016, Liu et al. [16] proposed that allergic lung inflammation aggravates Ang II-induced AAA and described that the allergic pneumonia promoted serum IgE that promoted inflammatory infiltration in AAA. Consistently, the importance of inflammatory infiltration in AAA pathology is also demonstrated in our study (Fig. 2A-F), and the important role for IgE in AAA is established in FcɛR1-knockout mice. Importantly, our study indicates that IgE promotes smooth muscle cell senescence, other than inflammation, to contribute to the formation of AAA.

SMCs play a crucial role in the development of AAA [21], in which SMC senescence contributes a large part of it [3, 22]. Chen et al. showed that vascular senescence led to AAA by reducing Sirtuin 1 [3]. Weiss proposed senescence was one of the risk factors associated with AAA [22]. Liao exhibited that AAA-derived SMC was more senescent than SMC from the adjacent inferior mesenteric artery [23]. However, it is lack of reports regarding the molecular mechanism mediating the senescence in SMC. In this study, we find that IgE up-regulates the expression of p21 via up-regulating LincRNA-p21 in SMCs, which promotes AAA via accelerating senescence. The expression of p53, the upstream factor of p21, is not altered with elevated IgE, suggesting that IgE induced lincRNA-p21 selectively targets p21.

LincRNA-p21 is a downstream long non-coding RNA transcript of p53. LincRNA-p21, as a repressor in p53-dependent transcriptional responses, participates in varied biological processes including apoptosis, metabolism, and senescence [15, 24]. It is not only limited to our research, but also in other studies, lincRNA-p21 plays an important role in cardiovascular diseases. Tang et al. demonstrated the first evidence that in Chinese Han population, the G-A-A-G haplotype of lincRNA-p21 decreased coronary artery disease and myocardial infarction risk [25]. Wu et al. identified lincRNA-p21 as a novel regulator of cell proliferation and apoptosis in atherosclerosis [15]. In this study, we also detected apoptosis of SMCs by different methods, including Flow cytometry, Western-blot and TUNEL Staining, which showed that IgE could induce the death of SMCs (Supplemetary Fig. 1A-C). When lincRNA-p21 was knocked down, IgE-induced apoptosis could be recovered partially (Supplemetary Fig. 1D). The rescue result was consistent with the results reported in the literature [15]. Nevertheless, we found that lincRNA-p21 accelerated the senescence of SMCs in AAA more evidently, demonstrating another function of lincRNA-p21 in cardiovascular diseases.

Overall, our study exhibits a novel molecular mechanism mediating the development of IgE promoted AAA. Likewise, asthma elevates serum-IgE that in turn up-regulates the expression of lincRNA-p21, resulting in up-regulation of p21 and senility of SMCs. As known, SMC senescence is proven to enhance the formation and rupture of AAA. Our result that siRNA against lincRNA-p21 blocked the IgE-induced SMC senescence, and significantly attenuated AAA provides a new therapeutic strategy for treating AAA.

## Supplemetary Materials

The Supplemenantry data can be found online at: www.aginganddisease.org/EN/10.14336/AD.2018.1128


