# Supplementary material for: IgE Aggravates the Senescence of Smooth Muscle Cells in Abdominal Aortic Aneurysm by Upregulating LincRNA-p21
Source: Aging Dis. 2019 Aug 1;10(4):699–710. doi: 10.14336/AD.2018.1128 (PMC6675527; doi:10.14336/AD.2018.1128)
Supplement: Supplementary file 1 [file AD-10-4-699-s.pdf]

SUPPLEMENTARY DATA

# IgE Aggravates the Senescence of Smooth Muscle Cells in Abdominal Aortic Aneurysm by Upregulating LincRNA-p21

**Wenjun Guo[#], Ran Gao[#], Wei Zhang[#], Weipeng Ge, Meng Ren, Bolun Li, Hongmei Zhao, Jing Wang[*]**

State Key Laboratory of Medical Molecular Biology, Institute of Basic Medical Sciences, Chinese Academy of Medical Sciences, Department of Pathophysiology, Peking Union Medical College, Beijing, China

© 2018. Guo W et al. Published online at http://www.aginganddisease.org/EN/10.14336/AD.2018.1128

# SUPPLEMENTARY DATA

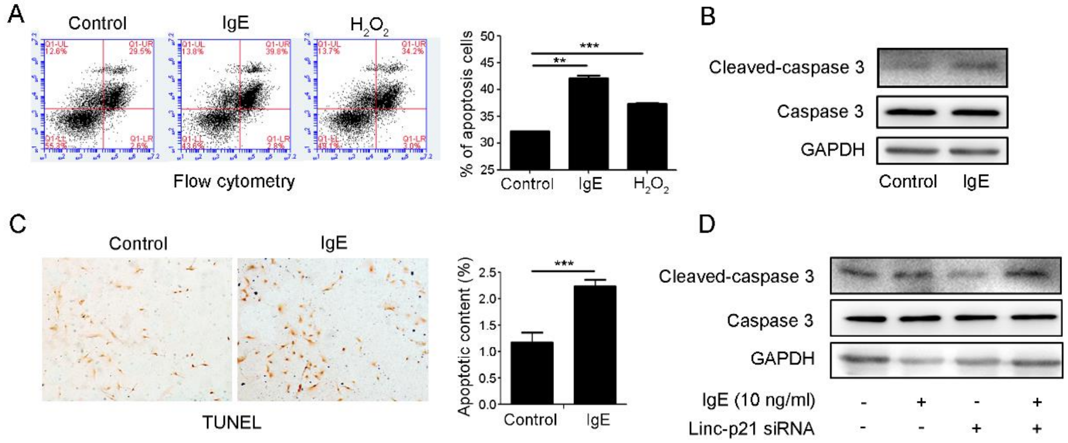


**Supplementary Figure 1. IgE effect on apoptosis of vascular SMCs *in vitro*.** (**A**) Flow cytometry detection of apoptosis of human vascular smooth muscle cells. (**B**) Western blotting analysis of cleaved-caspase 3 and caspase 3 expression in human vascular smooth muscle cells stimulated by IgE. (**C**) Representative images from TUNEL staining of human vascular smooth muscle cells. (**D**) Western blotting analysis of cleaved-caspase 3 and caspase 3 expression in human vascular smooth muscle cells stimulated by IgE and Linc-p21 siRNA. Data information: Data are presented as mean ± SEM. *P < 0.05; **P＜0.01; ***P＜0.001.

© 2018. Guo W et al. Published online at http://www.aginganddisease.org/EN/10.14336/AD.2018.1128
